# Malignant risk of thyroid nodules with isolated macrocalcifications – A study based on surgery results

**DOI:** 10.1016/j.clinsp.2025.100657

**Published:** 2025-04-24

**Authors:** Xi-Yue Yang, Li-Fang Huang, Yue-Jian Han, Xiao-Xin Cen

**Affiliations:** a Department of Diagnostic Ultrasound, Guigang People's Hospital, Guangxi, China; b Department of Pathology, Guigang People's Hospital, Guigang, Guangxi, China

**Keywords:** Thyroid nodules, Isolated macrocalcification, Malignant risk, Ultrasonography

## Abstract

**Objective:**

To determine the malignancy risk of thyroid nodules with Isolated Macrocalcifications (IMC) based on surgical results and evaluate the postoperative risk of malignant nodules with IMC.

**Methods:**

A total of 46 thyroid nodules with IMC were enrolled from 3680 consecutive patients who underwent thyroidectomy between August 2018 and September 2023. The malignancy risk of IMC nodules, postoperative risk of malignant nodules, and whether the ultrasonic features of IMC (smooth, lobulated, or focal disruption of the anterior margin) were associated with malignancy were investigated. The nodules were further divided into three groups (group A, maximum diameter < 10 mm; group B, maximum diameter of 10‒14 mm and group C, maximum diameter ≥ 15 mm). Differences in malignancy and Lymph Node Metastasis (LNM) risks were also evaluated among the three groups.

**Results:**

The malignancy risk of the IMC nodules was 30.43% (14/46). Four patients developed LNM. Eight nodules were staged as T1aN0M0 and low-risk, whereas six nodules were staged as T1bN1aM0 and intermediate-risk. Focal disruption of the anterior margin of IMC was significantly associated with malignancy. Malignant and LNM risk showed no differences among nodules with different sizes.

**Conclusions:**

IMC nodules with different sizes had a lower intermediate risk of malignancy and exhibited the same aggressive behavior. The cutoff value of these nodules for further Fine Needle Aspiration (FNA) warranted further investigation. Interruption of IMC was more often seen in malignant nodules, and more attention should be paid to these nodules.

## Highlights

• Focal disruption of the anterior margin of IMC was significantly associated with malignancy.
• Both small and large thyroid nodules with IMC had a lower intermediate risk of malignancy.
• Interruption of IMC is more often seen in malignant nodules.

## Introduction

Calcifications or echogenic foci of thyroid nodules are common on Ultrasound (US). It has been reported that up to 44.7 % of thyroid nodules present some kind of calcifications^1^ and 65.1 % of them are malignant.^2^ Calcifications are generally classified into microcalcifications (≤ 1 mm) and macrocalcifications (> 1 mm), according to their maximum diameters.^3^ It is well accepted that microcalcifications are often associated with malignancy, and various guidelines regard microcalcifications of thyroid nodules as suspicious malignant US features.^4^, ^5^, ^6^, ^7^, ^8^ However, the diagnostic value of macrocalcifications, particularly Isolated Macrocalcifications (IMC), remains controversial.^7^^,^^9^

IMC is a specific type of macrocalcification, defined as a calcified nodule accompanied by strong posterior acoustic shadowing, in which no soft tissue component is discriminated on the US.^10^ Several studies have reported that IMCs were only found in benign nodules,^9^^,^^11^ whereas two recent studies showed different results.^10^^,^^12^ A retrospective study based on the Fine-Needle Aspiration (FNA) showed that the malignancy rate of IMC is 18.4 %.^12^ Another study based on Core-Needle Biopsy (CNB) also demonstrated a medium malignancy rate in IMC.^10^ However, both FNA and CNB may yield false-negative results, particularly in nodules with macrocalcifications, which are harder and lack parenchyma.^13^^,^^14^ Further evidence is needed to establish the malignancy rate of IMC.

Therefore, the present study was conducted to determine the malignancy rate of IMC based on surgical results and evaluate the postoperative risk stratification of malignant IMC nodules.

## Material and methods

The Institutional Review Board of the Eight Affiliated Hospitals of Guangxi Medical University approved this retrospective study (GYYXLL-20,211,229–41) and waived the requirement for informed patient consent. The study followed the STROBE Statement.

### Study population

This retrospective study included 3680 consecutive patients who underwent partial or total thyroidectomy between August 2018 and September 2023 at the hospital. The inclusion criteria were as follows: 1) Patients who underwent US scans 3 months before surgery in the hospital and the stored US images could be clearly reviewed by sonologists; 2) Patients who underwent thyroidectomy and had a definite histopathologic diagnosis; 3) Patients with macrocalcification nodules, defined as calcified nodules accompanied by strong posterior acoustic shadowing and any soft tissue component that could not be distinguished on US; and 4) The number and location of nodules detected by US were in line with surgical findings.

### US exam and image analysis

**Fig. 1:**
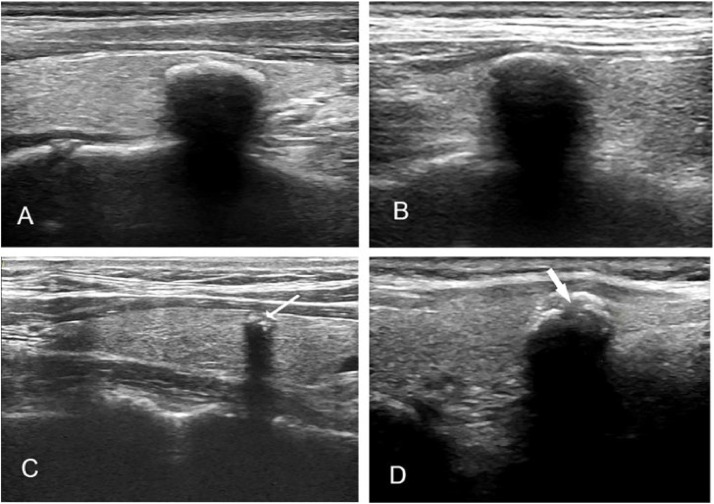
US features of IMC. (A) A benign IMC nodule with smooth margin in a 44 years old man; (B) A malignant IMC nodule with smooth margin in a 48 years old man; (C) A small (diameter = 5 mm) malignant IMC nodule with interruption of the anterior margin (arrow) in a 57 years old woman; (D) A large (diameter = 13 mm) malignant IMC nodule with interruption of the anterior margin (arrow) in a 47 years old woman.

US scans of the thyroid and neck were performed using a 5‒12 MHz linear array transducer (LOGIQ S8 or LOGIQ E9, GE Healthcare Wauwatosa, WI, USA). Sonograms of the nodules were preserved in a storage system, according to standard procedures. An experienced sonologist (Xiyue Yang) with 14 years of experience in the thyroid US retrospectively viewed the sonograms. The reviewer, who had no previous knowledge of the final diagnosis, determined the presence of IMC in the storage sonograms and assessed the US features of IMC, including whether the contour was smooth or lobulated or the presence of focal interruption (Fig. 1A–D). The nodules were divided into three groups according to the maximum diameter: group A, maximum diameter < 10 mm, group B, maximum diameter of 10‒14 mm and group C, maximum diameter ≥ 15 mm.

### Data analysis and statistics

The prevalence, malignancy rate, and size distribution of IMC were calculated. The postoperative cancer stage and clinical postoperative risk were also estimated using the eighth American Joint Committee on Cancer staging system^15^ and ATA risk stratification system.^4^ The authors also investigated whether the anterior margin of IMC was predictive of malignancy. Student's *t*-test or One-way ANOVA was used to compare differences in the measurement data. Chi-Square or Fisher's exact tests were used to determine the associations between categorical variables. The IBM SPSS Statistics for Windows software package ver. 21.0 (IBM Co., Armonk, NY, USA) was used to perform statistical analysis, and *p* < 0.05 was set as a significant difference.

## Results

### Demographic data

IMC was found in 46 of 3680 patients (1.25 %) who underwent thyroidectomy (35 women and 11 men; mean age, 50.5 ± 10.2 years, age range, 25–72 years). The nodule size ranged from 2 to 24 mm (mean size, 9.28±5.06 mm). Lymph Node Metastasis (LNM) was detected by preoperative US in two cases, and four cases were manifested after surgery in four be LNM. All malignant nodules were confirmed to be papillary thyroid carcinomas, and all benign nodules were nodular goiters.

### Malignancy risk of nodules with IMC

A final diagnosis was obtained for all 46 nodules, including 32 benign nodules (69.57 %) and 14 malignant nodules (30.43 %). Age, sex, and diameter of benign and malignant nodules were not statistically different (all *p* > 0.05).

There were 28 nodules in group A (maximum diameter < 10 mm), comprising 25.00 % (7/28) malignant nodules and 75.00 % (21/28) benign nodules. There were 12 nodules in group B (maximum diameter of 10‒14 mm), comprising 41.7 % (5/12) malignant nodules and 58.3 % (7/12) benign nodules. There were 6 nodules in group C (maximum diameter ≥ 15 mm), comprising 33.3 % (2/6) malignant nodules and 66.7 % (4/6) benign nodules. Neither age, sex, malignancy risk, nor LNM risk was significantly different among the three groups (Table 1).

**Table 1:** Comparison among the three groups [n ( %)].

	Age (yr)	Female ( %)	Malignancy risk ( %)	LNM risk ( %)
**Group (A)**	53.2 ± 9.2	20 (71.4)	7 (25.0)	1 (3.6)
**Group (B)**	47.9 ± 9.9	9 (75.0)	5 (41.7)	2 (16.7)
**Group (C)**	45.0 ± 9.5	6 (100)	2 (33.3)	1 (16.7)
χ**^2^/*F-*value**	2.601	‒	‒	‒
**p-value**	0.086^a^	0.471^b^	0.589^b^	0.194^b^

a One-way ANOVA.

b Fisher's exact test.

### Comparison of US features of IMC between benign and malignant nodules

Fisher's exact test showed that only the rate of focal disruption of calcification at the anterior margin was significantly different between benign and malignant nodules (3.12 % [1/32] vs. 42.86 % [6/14], *p* = 0.020). The rate of lobulated contours of the anterior margin showed no statistical difference between benign and malignant nodules (40.63 % [13/32] vs. 28.57 % [4/14]).

### Clinical features of the malignant nodules

Four nodules were LMN, and all were found in the central neck region. Three nodules with LNM were larger than 1 cm, and only one nodule was smaller than 1 cm. Eight nodules were staged as T1aN0M0 and low-risk, while the remaining six nodules were staged as T1bN1aM0 and intermediate-risk according to the Eighth American Joint Committee on Cancer staging system and ATA risk stratification system. No distant metastases were observed in the patients with malignant nodules.

## Discussion

The present study demonstrated that thyroid nodules with IMC had low to intermediate malignancy risks. Nodules with different sizes (maximum diameter < 10 mm, 10‒14 mm and ≥15 mm) had similar malignancy risks. Focal disruption of the anterior margin of calcification was significantly associated with malignancy.

The malignancy risk of thyroid nodules with IMC in the present study was 30.43 %, which was slightly higher than that reported by Na et al. (16.1 % and 23.3 % respectively).^10^^,^^12^ These discrepancies might be explained by the fact that their data were mostly based on FNA and/or CNB results, whereas this study was based on surgery results. It is well known that both FNA and CNB of thyroid nodules might yield a certain non-diagnostic and undetermined diagnosis,^16^^,^^17^ especially in nodules with macrocalcifications for needle movement, which might be restricted by hard calcification to acquire adequate cytological specimen.^14^ Similar to the study by Gwon et al.,^12^ the postoperative staging of the 14 nodules in this study presented a low to intermediate risk. The present study was based on histopathological results and provided more reliable information regarding thyroid nodules with IMC. To the best of our knowledge, this study is the first to evaluate the malignancy risk of IMC based on surgical results and to provide a more reliable reference for the management of IMC nodules.

The present results also showed that the malignancy risk was not significantly different among IMC nodules with different sizes. This finding was consistent with that reported by Na et al.^10^ Another study also showed that the malignancy rate of IMC nodules ≤15 mm was equal to that of nodules > 15 mm.^12^ According to the Korean Thyroid Imaging Reporting and Data System (K-TIRADS),^18^ the IMC nodules should be categorized as intermediate suspicious, and further FNA be taken when the nodule is larger than 10 mm. However, recent studies proposed that 15 mm be a better cutoff for further FNA for malignant risk was higher in nodules larger than 15 mm.^19^^,^^20^ Whereas, the malignant risk in this study showed no difference among nodules with different sizes. The small sample size of the present study might have resulted in the difference. As advocated by Sengul et al.,^21^, ^22^, ^23^ the cutoff size for further FNA of nodules with intermediate suspicious warranted further investigation.

Tumor size was considered an important factor to predictive cervical LNM in PTC, several studies reported that the larger the tumor the higher the LNM rate.^24^, ^25^, ^26^, ^27^, ^28^ However, the cutoffs for predicting LNM were still controversial. Many authors advocated 10 mm be the best cut-off,^25^^,^^27^, ^28^, ^29^ while a few studies reported that 2.5 mm or 5.0 mm would be better.^24^^,^^26^ The LNM rates among the three groups showed no significant difference in the present study which was more compared with the results of Yan et al. and Qu et al.^24^^,^^26^ which implies that both small and large nodules exhibit aggressive behavior. However, the retrospective design of the above studies (including the present study) might have yielded limited conclusions. A series of large prospective investigations are required to confirm if the tumor size plays a role as a prognostic factor in IMC nodules.

An irregular margin or interruption of macrocalcification often implies tumor invasion and is significantly associated with malignancy. The study conducted by Park et al. showed that the diagnostic rate of interrupted margins was as high as 75.0 %, with a specificity and negative positive value of 81.5 % and 88.5 %.^30^ A similar result was found in a study by Kim et al., which implied that macrocalcifications with irregular margins are often found in malignant nodules.^31^ The present study also showed that malignancy risk was significantly related to the anterior margin of calcification. This finding emphasizes that attention should be paid to IMC with irregular or interruption margins, and biopsy site selection at the interruption point would improve the diagnostic rate.^10^

The present study has several limitations. First, only patients who underwent surgery and those with confirmed pathological results were enrolled, which could have led to selection bias. Second, this retrospective study has some limitations in interpreting IMC. Third, this single-center study limited the sample size. Large-scale prospective studies are required to overcome these limitations.

## Conclusions

IMC nodules with different sizes have a lower intermediate risk of malignancy and exhibit the same aggressive behavior. The cutoff size of these nodules for further FNA warrants more investigations. Interruption of IMC is more often seen in malignant nodules, and more attention should be paid to these nodules.

## CRediT authorship contribution statement

**Xi-Yue Yang:** Conceptualization, Investigation, Writing – original draft. **Li-Fang Huang:** Methodology. **Yue-Jian Han:** Investigation, Data curation. **Xiao-Xin Cen:** Writing – review & editing.

## Conflicts of interest

The authors declare no conflicts of interest.
